# Tumour sampling method can significantly influence gene expression profiles derived from neoadjuvant window studies

**DOI:** 10.1038/srep29434

**Published:** 2016-07-07

**Authors:** Dominic A. Pearce, Laura M. Arthur, Arran K. Turnbull, Lorna Renshaw, Vicky S. Sabine, Jeremy S. Thomas, John M. S. Bartlett, J. Michael Dixon, Andrew H. Sims

**Affiliations:** 1 Edinburgh Cancer Research Centre, Institute of Genetics and Molecular Medicine, University of Edinburgh, Edinburgh, UK; 2 Ontario Institute for Cancer Research, Toronto, Ontario, Canada

## Abstract

Patient-matched transcriptomic studies using tumour samples before and after treatment allow inter-patient heterogeneity to be controlled, but tend not to include an untreated comparison. Here, Illumina BeadArray technology was used to measure dynamic changes in gene expression from thirty-seven paired diagnostic core and surgically excised breast cancer biopsies obtained from women receiving no treatment prior to surgery, to determine the impact of sampling method and tumour heterogeneity. Despite a lack of treatment and perhaps surprisingly, consistent changes in gene expression were identified during the diagnosis-surgery interval (48 up, 2 down; *Siggenes* FDR 0.05) in a manner independent of both subtype and sampling-interval length. Instead, tumour sampling method was seen to directly impact gene expression, with similar effects additionally identified in six published breast cancer datasets. In contrast with previous findings, our data does not support the concept of a significant wounding or immune response following biopsy in the absence of treatment and instead implicates a hypoxic response following the surgical biopsy. Whilst sampling-related gene expression changes are evident in treated samples, they are secondary to those associated with response to treatment. Nonetheless, sampling method remains a potential confounding factor for neoadjuvant study design.

Gene expression profiling of carcinomas has been widely used for molecular subtyping and prognostic prediction^1^,^2^,^3^,^4^,^5^,^6^,^7^,^8^,^9^ producing a diverse library of gene classifiers. However, these signatures may be limited by the particular dataset used to produce the signature^10^ and by the inherent cellular heterogeneity of tumours and the practical considerations of how samples are collected – in short the heterogeneity of the cohort and the heterogeneity of sampling.

A more informative and considered approach when performing molecular studies is to use matched biopsies from the same patient, allowing for both inter-patient variation to be controlled and changes occurring within a given tumour or organism to be more accurately modelled^11^,^12^,^13^. Matched sample pairs coupled with careful cohort selection should ensure that changes related to a given drug represent the largest source of variation, avoiding any unwanted contribution from confounding factors known or unknown and allow for greater statistical power with a smaller sample size^14^,^15^.

Acquisition of multiple biopsies from an individual patient has been simplified by the more common use of neoadjuvant therapy for breast cancer, an increasingly popular treatment option for initially large, inoperable or locally advanced breast tumours, as well as operable cancers susceptible to specific treatments^16^. Pre-operative treatment with chemotherapy, or endocrine therapy in ER+ disease, not only increases rates of breast conserving surgery^17^, but also allows a unique *in vivo* observation of tumour response to treatment^12^,^18^. This so-called ‘window of opportunity’ permits sequential biopsies of the same cancer to be taken at different time points during the course of the pre-operative treatment, allowing assessment of molecular changes in the tumour long before clinical evidence of response can be determined^11^,^13^. This has allowed the molecular effects of an administered drug to be studied and enabled biomarkers predictive of response or resistance to therapy to be identified at an increased rate. Recently our own group has demonstrated the added value of additional on-treatment measurements of gene expression to characterise and accurately predict the response to treatment^13^,^19^.

Whilst the benefits of this ‘window of opportunity’ approach are certainly attractive for translational research, matched samples have commonly been collected under the assumption that variation observed between pairs will have occurred as a result of treatment – i.e. the results apparent are due to the drug alone. A control group is often not included in these studies and those that do are commonly limited to a handful of samples (n = 8^20^, n = 15^21^) or are confounded by concurrent treatments. Conversely, studies that have compared multiple biopsies from the same patient in lieu of treatment are limited to only a fraction of the total molecular repertoire, most often focussing on hormone receptor status by IHC and not full transcriptomic profiling^22^,^23^,^24^,^25^. Whilst oestrogen receptor (ER), progesterone receptor (PR) and human epidermal growth factor receptor 2 (Her2) exhibit high concordance between sample pairs in these studies, the growing understanding of breast cancer as an increasingly heterogeneous and polygenic disease necessitates a high-throughput approach.

Previous work^26^ that has utilised larger-scale assays (a panel of 147 cancer related genes) investigated molecular variation under conditions of no-intervening treatment (NIT) in 21 paired core needle biopsy (CB) and excision biopsy (EB) samples. Here the diagnostic core biopsy was implicated in initiating an immune response, hypothesised to then be detected in a later surgically extracted excision biopsy. Potential stimulation of tumour-associated macrophages (TAMs) in response to CB was also reported, itself associated with poor prognosis in human breast cancer, raising concerns of taking multiple repeated biopsies from the same patient, underlining the importance of considering the full repertoire of genetic expression under conditions of no treatment.

Here we present the largest dataset to-date of untreated patient-matched breast cancer samples to determine whether, and to what extent, sample pairs exhibit molecular heterogeneity independent of treatment, and what the implications of any variation are in terms of the interpretation of patient-matched gene expression profiling studies. We explore possible causes of consistent differential expression and whether these reflect a wounding or immune response to the first biopsy, a hypoxia- or stress-induced response following blood supply interruption^27^ or the normal growth and evolution of tumours over time^28^.

## Methods

### Patient selection

Paired diagnostic core biopsies and surgical excision biopsies were identified from 37 patients with a primary histologically confirmed invasive breast cancer that did not receive any preoperative or neoadjuvant treatment at the Western General Hospital, Edinburgh, between 2003 and 2011. All patients gave informed consent to be included in the study, which was approved by the Lothian Regional Ethics Committee (2001/8/80 and 2001/8/81) and we confirm can that all experiments were performed in accordance with relevant guidelines and regulations. A summary and complete clinicopathological characteristics of the patients diagnostic core biopsies are given in Supplementary Tables S1 and S2.

### Sample collection, RNA processing and microarray hybridisation

Core biopsies were taken at diagnosis in all patients using a 14-gauge automated needle device. Multiple cores were taken per tumour and combined as individual samples. Surgical excision biopsies of breast tumour were collected between 13 and 53 days later (mean interval = 27.5 days). Samples were snap frozen in liquid nitrogen and stored at −80 °C before homogenisation and RNA extraction using the RNeasy Mini Kit with RNAse Free DNAse treatment (Qiagen). RNA quantity and quality was assured using a Nanodrop 2000 c spectrophotometer (Thermo Scientific). RNA was reverse transcribed and amplified using the WT-Ovation FFPE System Version 2 (NuGEN), purified using the Qiaquick PCR purification Kit (NuGEN), biotinylated using the IL Encore Biotin Module (NuGEN), and purified using the minElute Reaction Cleanup Kit (Qiagen). At each step RNA/cDNA quantity and quality was assured by repeat assessment with the Nanodrop 2000 c prior to advancing to the next stage. Labelled cDNA was hybridized to Illumina Human HT-12 version 3 and version 4 whole-genome expression bead arrays according to the standard protocol for NuGEN labelled samples. Data was extracted using GenomeStudio software (Illumina).

### Data analysis

All analysis was conducted in R (http://www.r-project.org) using software packages available via CRAN (http://cran.r-project.org/) and Bioconductor (http://www.bioconductor.org/). Data was pre-processed using the *lumi* package^29^. Log2 transformation, quantile normalisation and summarisation was performed for all Illumina probe profiles. Probe expression information was extracted and detected probes were standardised, firstly by passing a detection p-value threshold (≤0.05) and then by being called present in ≥3 samples. This was carried out three times, once each for three processing batches that comprised the dataset. A common feature list was determined by those probes common to all three dataset batches before re-mapping to Ensembl gene sequences using the *biomaRt* package^30^,^31^. Multiple probes-per-Ensembl ID were resolved by mean averaging. Batch correction was performed using ComBat^32^ to integrate processing batches for further analysis (Supplementary Fig. S2). Sample groupings were compared using Pearson product-moment correlations. All significances were calculated by a two-tailed Wilcoxon rank sum test and corrected for multiple testing (FDR), unless otherwise stated. Intrinsic subtype assignment was performed using the *genefu* package^33^. Pairwise differential gene expression was calculated using significance analysis of microarrays (SAM), part of the *siggenes* package^34^. Hierarchical clustering was performed on pairwise fold change expression values using a complete linkage method. SAM analysis of treated data was performed using a hundred 18-sample permutations, to fairly match sample size between subsets, with the intersecting significant genes being taken as a mean average. The raw and processed data from this study can be accessed from NCBI GEO under the accession GSE76728.

## Results

### Consistent treatment-independent gene expression changes between diagnostic and surgical paired samples

**Figure 1:**
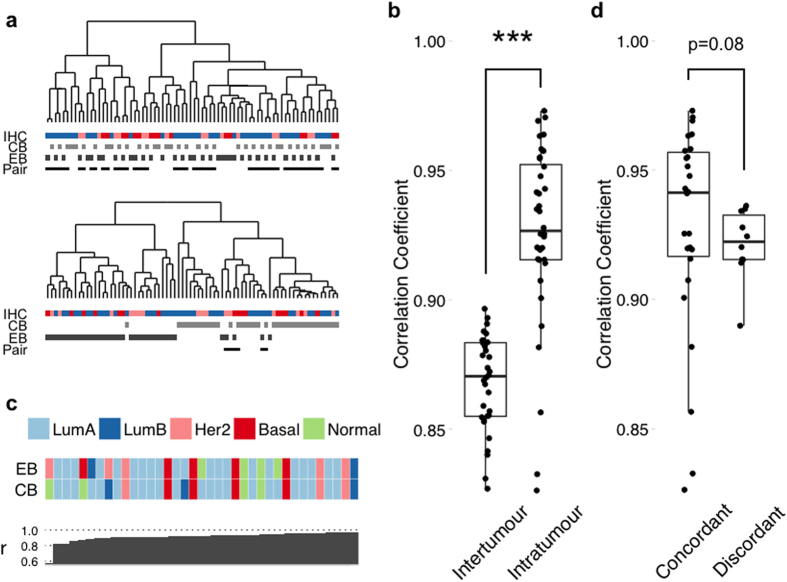
Evidence of treatment independent variation between breast cancer diagnostic core biopsies and surgical excision samples. (**a**) Hierarchical clustering of the 37 patient-matched diagnostic core and excision biopsy samples using the 500 most variable genes (upper) and a SAM derived signature of 50 genes consistently differentially expressed between core and excision biopsies (lower). Bars represent IHC status (ER+/Her2− = Blue; ER+/Her2+ = Pink; ER-/Her2− = Red) or biopsy method. Lower-most bar indicates where sample pairs have co-aggregated. Two-thirds (25/37) of the pairs cluster at the first level of the upper dendrogram, whereas pairwise association is lost in 31/37 cases for the lower. (**b**) There is a significantly stronger correlation between biopsy pairs (intra-tumour) than between different tumours (mean inter-tumour). ***p < 0.001. (**c**) Discordance in molecular subtype assignment between core and excision biopsies. Patients are ranked left to right by pairwise correlation. Colours represent SSP subtypes (Luminal A = Dark blue; Luminal B = Light Blue; Her2 = Pink; Basal = Red; Normal = Green). (**d**) Sample pairs were called discordant when Biopsy A ≠Biopsy B for at least 4/5 classifiers. Comparison of concordant vs. discordant pairwise correlations then revealed an inverse relationship between correlation and discordancy.

Unsupervised clustering using the 500 most variable genes across the patient-matched samples demonstrated high concordance, with 25/37 pairs observed to cluster at the first level of the dendrogram (Fig. 1a upper). In order to assess whether core biopsy and surgical excision sample pairs varied from one another, Pearson correlations were calculated between intra-tumour (paired) and inter-tumour (non-paired) samples (Fig. 1B). Intra-tumour differences were more significantly (p = 7e-11) correlated (median r = 0.92 range r = 0.60–0.97) than the mean inter-tumour variations (median = 0.87, range r = 0.77–0.90). Technical interference due to sample processing is expected to decrease correlation between samples, though only nominally^35^. Correlations below r ≃ 0.97 are likely due to underlying tumour heterogeneity, implicating a biological cause to the variation.

To determine the impact of differences in gene expression apparent between diagnostic core and excision biopsy pairs, pairwise SAM analysis identified 50 significantly differentially expressed genes (48 upregulated, 2 downregulated; FDR = 5%), between the surgical excision and diagnostic core biopsies (Supplementary Table S3). Clustering using these genes was able to separate samples by their sampling method in 31/37 cases (Fig. 1a lower). Five subtyping signatures (PAM50, Sorlie 2003, Hu 2006, Desmedt 2008, Wirapati 2008) were applied to each sample individually and concordant (subtype agreement between pairs with >3 subtype classifiers) vs. discordant (≤3 pair subtype agreements) results recorded (Fig. 1c (PAM50 only) and S5 (all methods)). Pairwise correlation between concordant and discordant assignments, revealed a trend (p = 0.08, Wilcoxon) between decreased pairwise correlation and greater discordance (Fig. 1d).

Pathway analysis of the 50 gene NIT signature revealed enrichment for MAPK signalling (DUSP1, JUN, NR4A1 and FOS), cancer specific (COX-2, PGE2, JUN and FOS), apoptosis induction (FOS and JUN) and genomic reformatting following (brain) ischaemia (EGR1 and JUN) pathways. A number of these genes are examples of ‘early’ or ‘primary’ growth response genes induced by both cell-extrinsic and cell intrinsic signals that do not require *de novo* protein synthesis for their expression^36^.

### Patient-matched gene expression changes may be associated with either time or biopsy methodology

**Figure 2:**
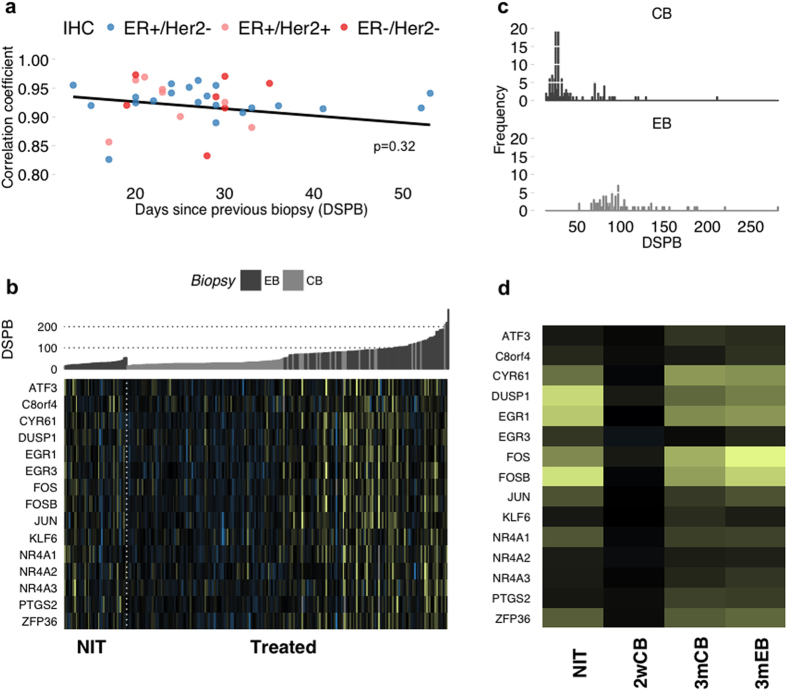
Factors associated with consistent gene expression changes between diagnostic core biopsy and surgical excision of breast tumours in the absence of treatment. (**a**) Pairwise correlations between biopsy pairs are not explained as either a function of time between biopsy (p = 0.32) or IHC status (p = 0.43). ER+/Her2− = Blue; ER+/Her2+ = Pink; ER-/Her2− = Red. (**b**) Heatmap showing differential expression of NIT signature genes in NIT and letrozole treated cohorts. Colours represent gene expression fold changes (up = yellow; down = blue) between samples and their subsequent patient-matched biopsies. Samples are ordered by increasing time between biopsies and reveal a pattern associated with either extraction method - CB (grey) or EB (dark grey) - or time. (**c**) Frequency distribution of biopsy time intervals. For further analysis, the letrozole treated data was split into three subsets – 2-week CB (2wCB), 3-month CB (3 mCB) and 3-month EB (3 mEB) – to investigate the effects of biopsy method and/or time on gene expression. (**d**) Mean expression fold changes since previous biopsy for 2 week, 3 month and NIT samples. 3 month subsets closely resemble NIT expression changes, though SAM analysis revealed a greater intersection of differentially expressed genes between NIT and 3 mEB samples than between NIT and 3 mCB samples.

Having determined significant and consistent changes in gene expression exist between diagnostic core and surgical excision pairs we sought to identify the underlying cause. Several hypotheses were immediately apparent – greater changes may occur following a shorter time interval between sampling if consistent gene expression changes reflect a wounding/immune response to the diagnostic core biopsy^26^; or it may be anticipated that expression patterns diverge over time to reflect tumour evolution; this may in turn be driven by tumour subtype^28^. However, comparisons of pairwise correlations defined by either IHC status subtype (ER+/Her2−, ER+/HER2+ or ER-/HER2−), PAM50 subtype (cross-table comparing IHC and PAM50 subtype assignment in Supplementary Table S4) or as a function of time interval between biopsies revealed no trend associated with either factor (p = 0.43 and p = 0.32 respectively) (Fig. 2a). This suggested that a progression in breast cancer-related biological changes were unlikely to be the root cause of the observed pairwise variation. To further investigate whether breast cancer biology could be responsible for the observed pairwise variation, we compared 7 breast cancer-related expression modules^37^ between core and excision biopsies (Supplementary Fig. S3), which revealed no contrasting trend in gene expression. In conjunction to assigning cause, it remained equally important to determine whether our NIT signature genes were evident to alter in an equivalent treated data set. Using a patient-matched cohort treated with letrozole, collected and processed within our group in a manner analogous to the NIT data^13^, there appeared to be a strong relationship between the time interval between biopsies and a subset of our NIT gene signature (Figs 2b and S4), with treated samples biopsied after 3 months exhibiting significant differential expression of these NIT genes. In these instances however, time interval almost wholly coincided with the final biopsy method and we therefore sought to determine which factor, if either, was dominant. Dividing the treated data into 3 subsets determined by the time since previous biopsy (Fig. 2c) as 2-week CB (2wCB, n = 95), 3-month CB (3 mCB, n = 18) and 3-month EB (3 mEB, n = 70), allowed comparison of changes assumed to result from treatment by both time interval and sampling method. Biopsy time interval was apparent as the factor most associated with the genes altered in our NIT data, with 3-month samples alone exhibiting expression fold changes similar to those observed in the NIT data, implying time on treatment as the defining factor (Fig. 2d). However, in tandem, global GSEA analysis was able to demonstrate that our NIT signature could significantly define the differences between untreated and treated data only when sampling method differed (NIT vs. 2wCB, p = 0; NIT vs. 3 mCB, p = 0.02; NIT vs. 3 mEB, p = 0.25). Similarly, only in the instance of excision biopsy was SAM analysis able to recapitulate >30% of the genes differentially expressed in the NIT data (mean = 47%). More so, out of 3955 differentially expressed treated-EB genes, seven of those common to NIT differential expression were amongst the top 15 in terms of fold change magnitude. Taken together, this implies both time on treatment as well as biopsy method are able to impact upon NIT signature gene expression.

### Multiple patient-matched datasets also demonstrate changes in NIT early growth response genes

**Figure 3:**
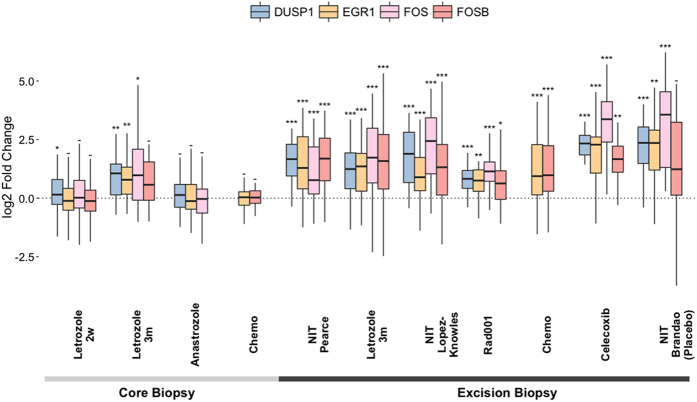
Multiple patient-matched datasets demonstrate shared changes in NIT early growth response genes. Pairwise analysis of four early growth response genes among the NIT signature in six validation datasets. These genes potentially represent an association between gene expression and sampling method, with surgically excised samples (EB) showing greater expression fold-change than their core biopsied (CB) counterparts. *p ≤ 0.05; **p ≤ 0.01; ***p ≤ 0.001; −** **= not significant.

To further investigate the evident relationship between sampling method, time on treatment and NIT signature expression, we selected a panel of four genes (DUSP1, EGR1, FOS, FOSB) that were observed to be most representative of these factors, as well as being well characterised in the literature, for comparison in six external validation datasets (Table 1, Fig. 3). Significant differential gene expression was consistently observed to a greater degree only when an excision biopsy followed a previous core biopsy for these four genes.

### Tumour sampling appears independent of an immune or wound-healing response

It remained important to determine whether gene expression changes in lieu of treatment were able to elicit either an immune or wound-healing response that may be detrimental to a patient’s health or directly confound gene expression profiling results. Evaluation of both a published immune-related 9 gene panel, suggested to be upregulated in response to a diagnostic CB^26^ and a 589 gene signature representative of a wound-healing response^38^ failed to show any association with sampling method or time between biopsy, with sample expression unlikely to have been affected in terms of these biological categories (Supplementary Fig. S2).

## Discussion

Our study reports gene expression changes in the largest cohort of sequential samples from patients receiving no-intervening treatment yet assembled to demonstrate the molecular variation that occurs independent of treatment in the neoadjuvant and preoperative setting. Significant pairwise changes in gene expression were observed and a 50 gene signature identified comprised of genes associated with a number of cell growth, cell stress and cancer related signalling pathways, including ATF3, EGR1, FOS, FOSB and JUN, each of which have been previously implicated in prognostic discrimination and pathogenesis of breast cancer^39^,^40^,^41^,^42^,^43^ as well as other cancers^44^.

We report that sampling method, specifically core versus excision biopsy, has a direct impact on gene expression and has the potential to introduce a confounding factor to downstream analysis. The most probable explanation of this expression variation between sample pairs is the technical issue of warm ischemia before newly biopsied samples are processed, when the cellular metabolic machinery attempts to mount a survival or apoptotic response before all metabolic activity ceases. Tissue ischemia may result from exclusion of the vascular supply or simply from handling by the surgeon, scrub nurse, pathologist and tumour bank personnel before the sample is frozen in liquid nitrogen. This time delay is likely (on average) to be significantly longer for surgical excision specimens than core biopsy samples. The effects of ischemia on gene expression has been described previously^45^ and warm ischemia associated with the surgical extirpation of human tissues has significant effects on gene expression. These data support the careful monitoring of ischemic time for tissues harvested for the purpose of gene profiling. Similarly, Dash *et al.*^46^ demonstrated significant changes in the expression of FOS, JUN, ATF3 in a study to examine the effect of processing time on prostate cancer samples.

Similar conclusions are proposed in a recent study published during the review process^47^, where CB and EB pairs were also analysed in terms of correlation and differential gene expression. Variation between sample pairs was found to be evident though modest and those genes found to be differentially expressed intersected with those highlighted in this study (34% of our NIT signature genes). Of particular interest, the overlapping genes included the four genes highlighted in Fig. 3 as well as several others indicative of a stress or early growth response (RGS1, RGS2, ATF3, JUN), similarly proposed as a reaction to surgery-associated ischaemia. Indeed, the rationale of the study stemmed from a desire to investigate post-operative processing time and its effect on gene expression, citing two smaller studies which both highlighted ischaemia as a potential source of molecular variation^48^,^49^. Again, key molecules including FOSB were highlighted as being demonstrative of this effect. Importantly, the investigators additionally compared excision biopsies placed into RNAlater either immediately post-surgery or following an interval, and again observed early growth and stress response associated genetic expression to increase.

In a second analogous study, of 147 breast cancer-related genes measured by Nanostring in 21 patients, Jeselsohn *et al.*^26^ proposed that sequential breast cancer biopsies reveal activation of an immune response, characterised by a panel of 9 immune-related genes, of which CD68 is known to activate tumour-associated macrophages and implicated in increasingly severe prognosis^50^, as well as being present in the clinically available Onco*type* DX^®^ breast cancer assay^8^. However, none of these genes were found to be significantly differentially expressed in our larger, whole genome cohort (Supplementary Fig. S1). Conversely, a number of studies have demonstrated a high degree of concordance between classical IHC markers for breast cancer, namely oestrogen, progesterone and HER2 receptors^24^,^25^ between diagnostic core and excision biopsies, suggesting discordance may be limited to the level of transcription. Our study demonstrates that pairwise variation at the transcriptome level is not limited to the classical markers of breast cancer, though in some cases paired samples may be classified differently using molecular signatures (Figs 1c and S5). The causative factor behind this variation is most likely due to sampling method in our NIT cohort, with surgical resection resulting in gene expression in response to stress. It remains unclear whether this effect translates to samples following treatment, with time on treatment being observed to mediate molecular changes against the background of the sampling method.

It is important that results from preoperative window or neoadjuvant studies are carefully scrutinised, as a study by Morrogh *et al.*^51^ - using a 502 cancer-related gene panel to examine 16 paired patient samples, 8 of which were untreated controls - claimed that window trials are influenced by the wound-healing process. They proposed that upregulation of MLL and FOSB was evidence of this, irrespective of treatment. This mirrored our own findings, though it is necessary to note that Morrogh *et al.* were limited by sample size and inconsistencies in the effect direction of proliferation markers (increased Ki67, but decreased PCNA). Nonetheless it remains likely that the overall pathway level message within our study - upregulation of proliferation - was a consequence of an early growth response to the biopsy itself. It is crucial to determine whether there is a genuine immune response associated with biopsy type as inflammatory signatures have been associated with poor anti-proliferative responses to aromatase inhibitors^13^,^52^. Clear evidence of both an immune or wound response signature^53^ was completely absent in our untreated samples, implying that any contribution of biopsy methodology to an inflammatory response is likely to be minor.

With the potential for pairwise variation irrespective of treatment, our study raises potential concerns of the suitability of the neoadjuvant window in gene expression profiling studies. Recent results of the ALTTO (Adjuvant Lapatinib And/Or Trastuzumab Treatment Optimisation) clinical trial were, however, found to be consistent with the predicted benefits from the neoALTTO trial. This supported the utility of the neoadjuvant setting as a suitable and important window for evaluating promising new targeted agents^54^, as well as the continued use of patient-matched samples to assess intervention studies for translational research. Nonetheless it remains critical to understand that whilst patient-matched samples reduce variation due to individuals, all possible sources of variation must be considered for an optimal experimental design. For example, in an intervention study to assess dietary changes on normal breast tissue from pre-menopausal women it was considered optimal to schedule the sequential biopsies one menstrual cycle apart, rather than using a fixed window of time^55^, as there is clear evidence that menstrual changes in oestrogen levels caused significant changes in gene expression^56^. Underlying tumour heterogeneity is an inevitable variable when performing neoadjuvant or window studies, however our study suggests that the method of sample collection should be considered along with treatment, time interval and clinicopathological features as an important potential confounding factor. These considerations are of particular importance if a study’s purpose is the development of a prognostic/predictive classifier or identification of a biomarker, with genes present in our NIT signature excluded from the analysis.

Our study demonstrates that consistent molecular changes arise in tumours in the absence of treatment and these can impact upon classification. These changes appear to be an artefactual ischemic response resulting from the sampling methodology itself, rather than reflecting the effects of a previous biopsy. Careful consideration should be given in future studies that seek to illustrate molecular changes between paired biopsies in the neoadjuvant setting for breast cancer and likely other cancers that make use of the same experimental design.

## Additional Information

**Accession Code**: The raw and processed data from this study can be accessed from NCBI GEO under the accession GSE76728.

**How to cite this article**: Pearce, D. A. *et al.* Tumour sampling method can significantly influence gene expression profiles derived from neoadjuvant window studies. *Sci. Rep.* **6**, 29434; doi: 10.1038/srep29434 (2016).

## Supplementary Material

Supplementary Information

## Figures and Tables

**Table 1:** Composition of patient-matched neoadjuvant breast tumour datasets used within our study.

Neoadjuvant treatment	n	Biopsy Time Interval/days		Extraction Method			Dataset/Reference
		median	range	1^st^	On-	Final	
None	37	27	13–53	CB	–	EB	This study
Letrozole	122	107	13–884	CB	CB	CB EB	Turnbull *et al.*^13^
Celecoxib/none	22 15	*unspecified*	14–21	CB	–	EB	(Brandão *et al.*^21^
Anastrozole	81 On-18 Final	14	14–112	CB	CB	CB	(Smith *et al.*^57^
RAD001	21	14	14	CB	–	EB	(Sabine *et al.*^58^
Anthracycline-based Chemotherapy	69	*unspecified*	*unspecified*	CB	CB	EB	(Magbanua *et al.*^59^
None	56	*14*	*14*	CB		EB	Lopez-Knowles *et al.*^47^
