# Supplementary material for: Tumour sampling method can significantly influence gene expression profiles derived from neoadjuvant window studies
Source: Sci Rep. 2016 Jul 7;6:29434. doi: 10.1038/srep29434 (PMC4935948; doi:10.1038/srep29434)
Supplement: Supplementary Information [file srep29434-s1.pdf]

# Supplementary Data for:

## Tumour sampling method can significantly influence gene expression profiles derived from neoadjuvant window studies

Dominic A. Pearce[1], Laura M. Arthur[1], Arran K. Turnbull[1], Lorna Renshaw[1], Vicky S. Sabine[1,2], Jeremy S. Thomas[1], John M. S. Bartlett[1,2], J. Michael Dixon[1], Andrew H. Sims*[1].

[1]Edinburgh Cancer Research Centre, Institute of Genetics and Molecular Medicine, University of Edinburgh, Edinburgh, UK

[2]Ontario Institute for Cancer Research, Toronto, Ontario, Canada

*corresponding author andrew.sims@ed.ac.uk

**Contents:**

**Supplementary Figures S1-S5**

**Supplementary Tables S1-S4**

**Supplementary Data:**

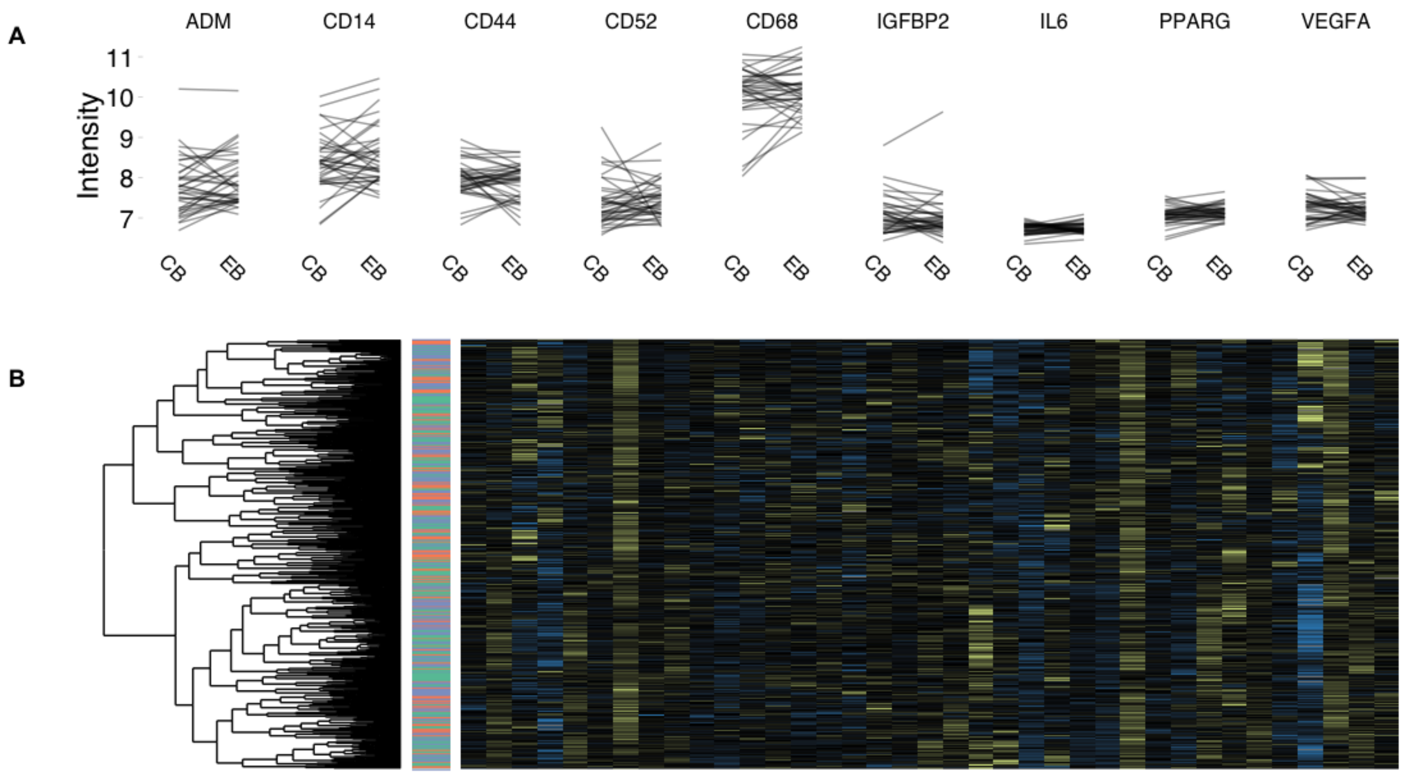


**Supplementary Figure S1. Tumour sampling is independent of an immune- or wound-healing response.** Top, Pairwise expression changes for 9 immune-related genes, reported by Jeselsohn et al. [1], between CB and EB NIT samples. Changes are observed to lack clear effect direction with 0/9 found to be significantly differentially regulated. Bottom, Heatmap detailing differential expression of a 589 gene wound-healing signature in NIT data, ordered by increasing biopsy time interval (Yellow = high expression, blue = low expression). Colour bar represents signature gene class – activated (blue), quiescent (green) or cell cycle (orange). Lack of any coherent gene clustering by class implies the absence of a true wound-healing response.

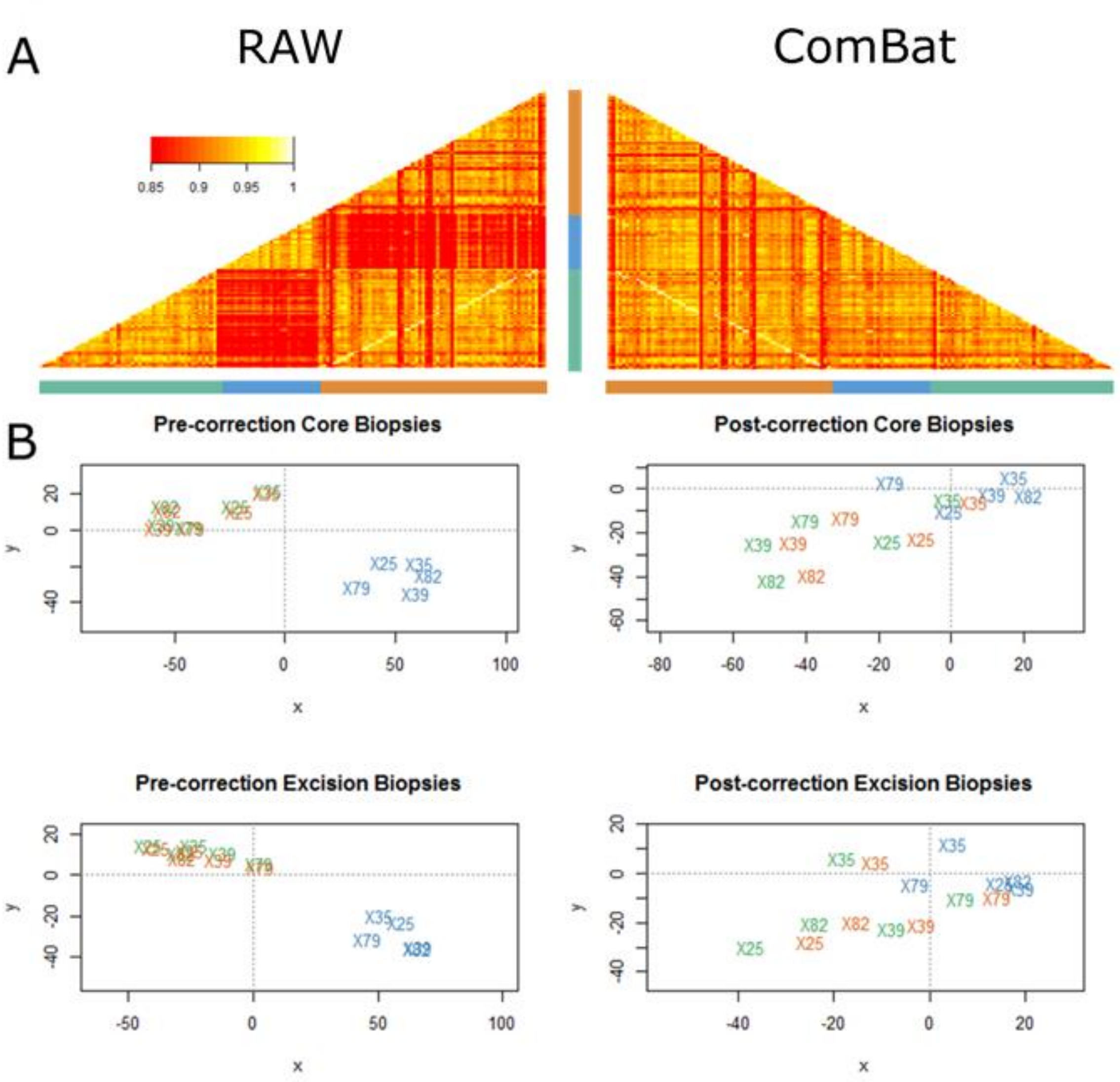


**Supplementary Figure S2. Correction for dataset batch effects.** RNA extraction and preparation prior to microarray hybridisation was performed using identical protocols in a single centre. However, Datasets 1 (orange) and 3 (green) were hybridised to Illumina Human HT-12 version 4 whole-genome expression bead arrays, whereas Dataset 2 (blue) hybridisation was performed on version 3 of the same platform. This resulted in an observable batch effect between uncorrected Dataset 2 and Datasets 1 & 3, when comparing sample Pearson correlations by correlation heatmap (A) or multi-dimensional scaling (B). The ComBat method was applied to remove batch effects and facilitate robust dataset integration.

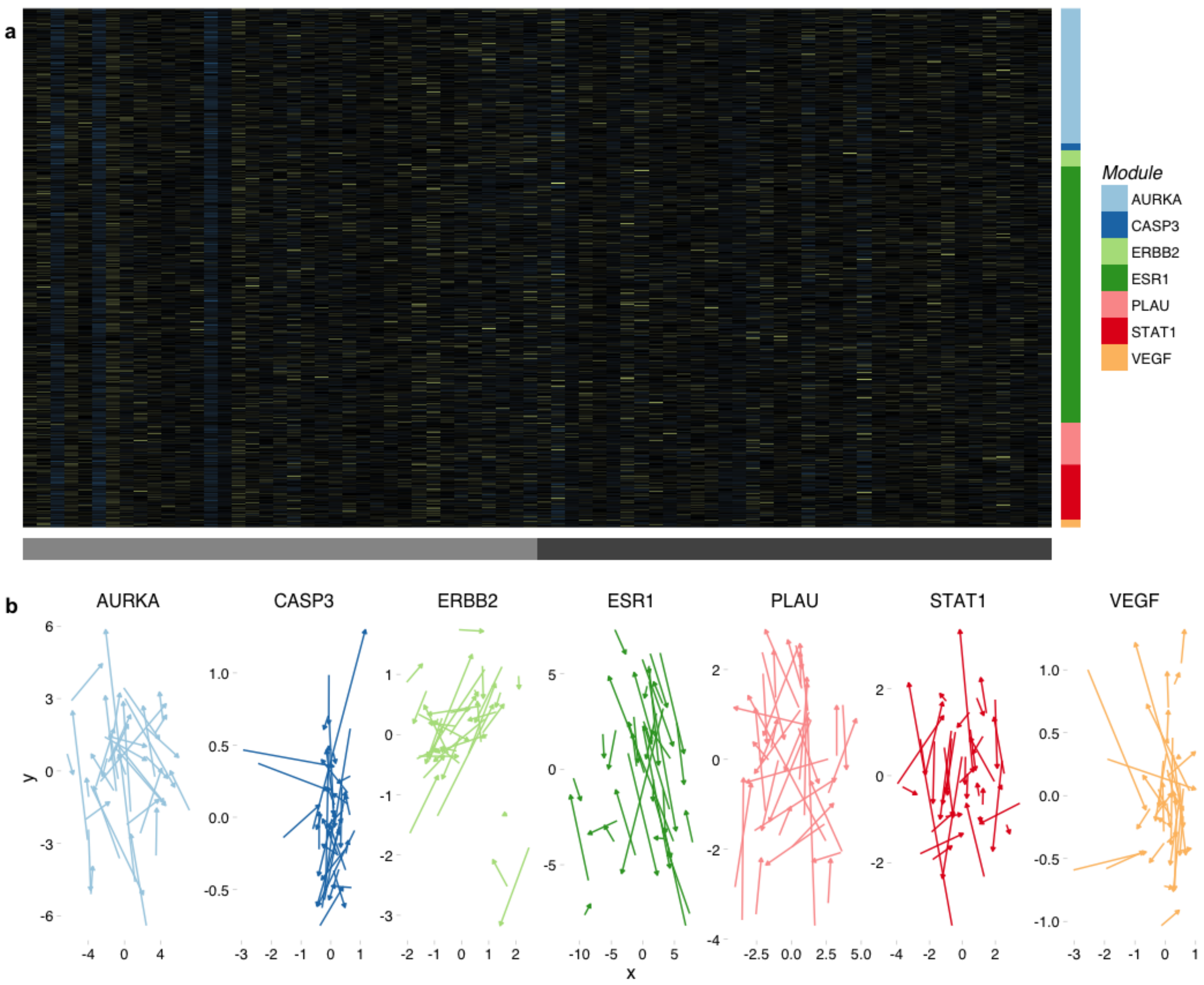


**Supplementary Figure S3. Effects of tumour sampling on 7 breast cancer-related expression modules.** Seven gene expression modules defined by Desmedt et al.[1] were investigated to determine whether tumour sampling could affect breast cancer-related processes. (a) A heatmap compares expression (yellow = greater than row mean expression, blue = lesser than row mean expression) of core biopsied samples (light grey) with excision biopsied samples (dark grey) for the different modules (y-axis colour bar). (b) Pairwise analysis of module expression by MDS. Patient samples display a lack of uniform movement or direction as they transition from core biopsy (arrow tail) to excision biopsy (arrow head), implying a lack of a systematic breast cancer biology-related effect.

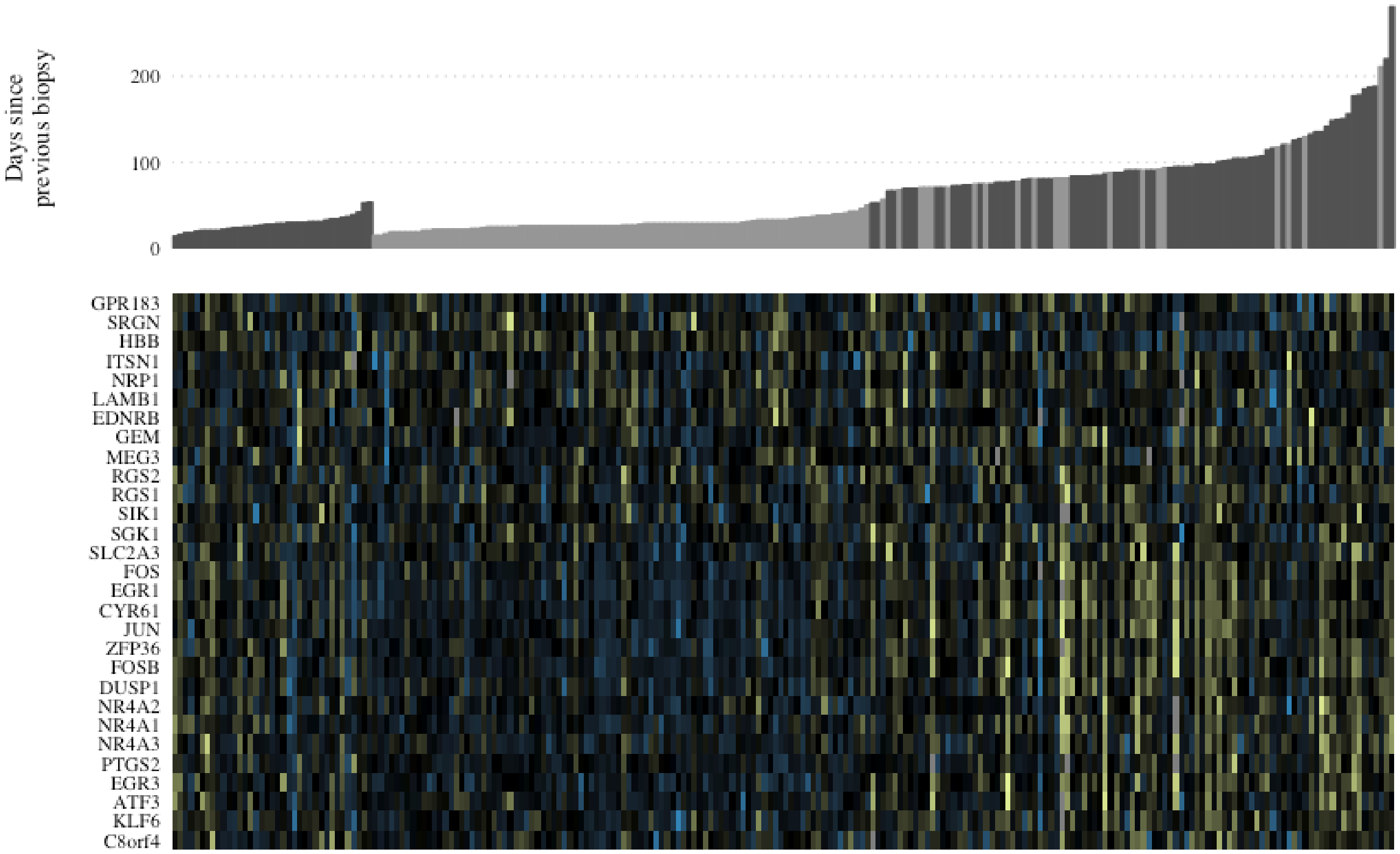


**Supplementary Figure S4. Heatmap showing differential expression of NIT signature genes in NIT and letrozole treated cohorts.** Colours represent gene expression fold changes (up = yellow; down = blue) between samples and their subsequent patient-matched biopsies. Samples are ordered by increasing time between biopsies. Core biopsy = grey; Excision biopsy = dark grey.

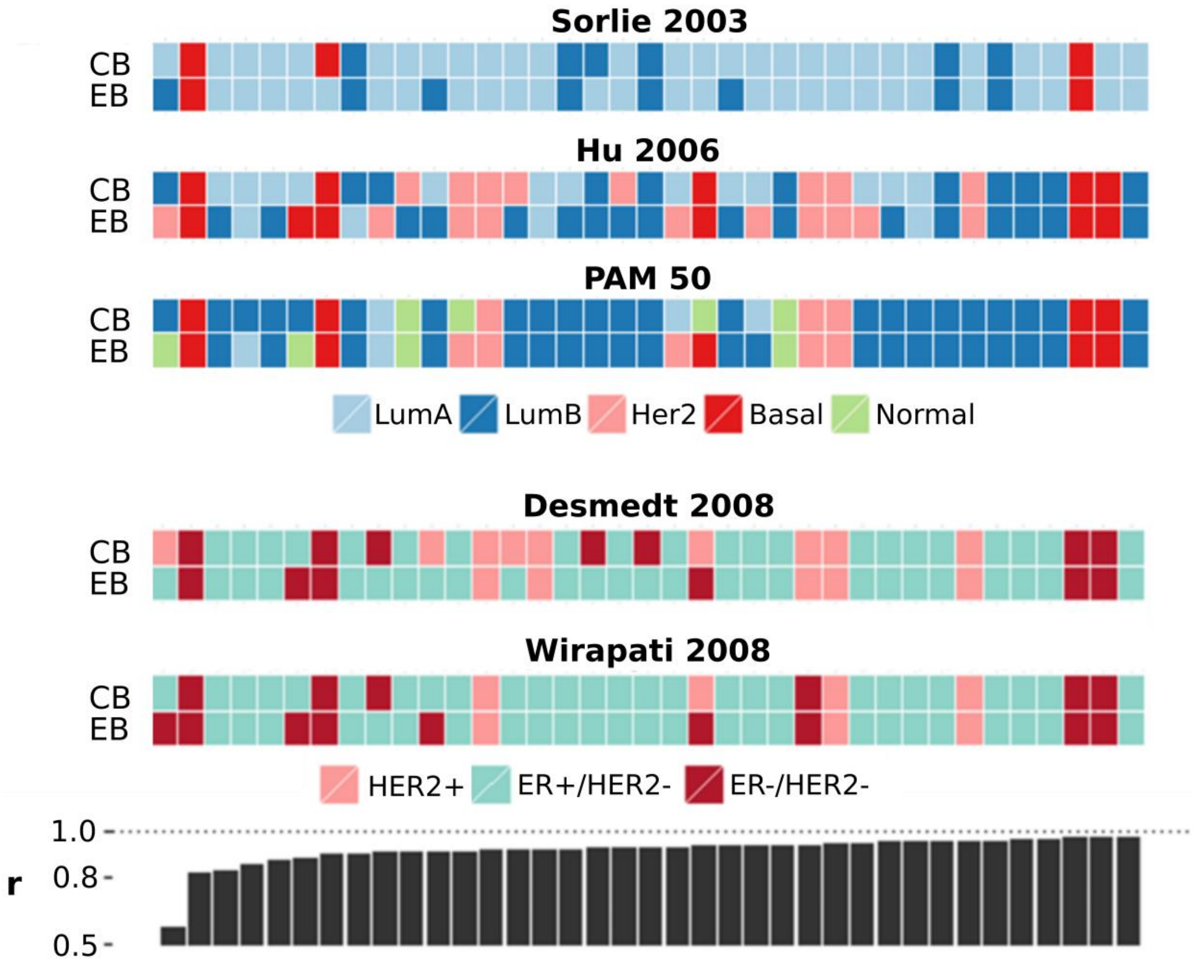


**Supplementary Figure S5. Discordance in molecular subtype assignment between core and excision biopsies.** Patients are ranked left to right by pairwise correlation. Colours represent SSP (Luminal A = Dark blue; Luminal B = Light Blue; Her2 = Pink; Basal = Red; Normal = Green) and SCM (HER2+ = Pink; ER+/HER2- = Turquoise; ER-/HER2- = Burgundy) subtypes.

**Supplementary Table S1 - Summary clinicopathological features of the tumours in the study**

| **IHC Status** | **No. Patients** |
|---|---|
| *ER+/Her2-* | 22 (~59%) |
| *ER+/Her2+* | 8 (~21%) |
| *ER-/Her2-* | 7 (~19%) |
| **Size (mm)** | |
| *≤20* | 12 (~32%) |
| *21-50* | 21 (~57%) |
| *51-60* | 2 (~5%) |
| *NA* | 2 (~5%) |
| **Grade (Elston-Ellis)** | |
| *1* | 3 (~8%) |
| *2* | 15 (~41%) |
| *3* | 18 (~49%) |
| *NA* | 1 (~3%) |
| **Nodal Involvement** | |
| *Positive* | 18 (~49%) |
| *Negative* | 19 (~51%) |
| **Age (years)** | |
| *≤40* | 5 (~ 14%) |
| *41-50* | 5 (~ 14%) |
| *51-60* | 5 (~ 14%) |
| *61-70* | 8 (~22%) |
| *70-80* | 9 (~24%) |
| *80+* | 5 (~ 14%) |
| **Final Biopsy** | |
| *CB* | 0 (0%) |
| *EB* | 37 (100%) |

**Supplementary Table S2 - Complete clinicopathological features of the tumours in the study**

| StudyID | Grade (Elston-Ellis) | Size (mm) | ER (Allred) | PR | Her 2 | Nodes |
|---|---|---|---|---|---|---|
| 1 | 3 | 33 | 0 | 0 | 2+ FISH-ve | Neg |
| 2 | 1 | 38 | 7 | NA | 2+FISH-ve | Pos |
| 3 | 3 | 47 | 6 | NA | 2+FISH-ve | Pos |
| 4 | 2 | 29 | 8 | NA | 2+FISH-ve | Neg |
| 5 | 3 | 14 | 7 | 5 | 3+ | Neg |
| 6 | 2 | 29 | 8 | NA | 1+ | Pos |
| 7 | 3 | 31 | 3 | 0 | 2+FISH-ve | Pos |
| 8 | 3 | 15 | 8 | NA | 3+ | Neg |
| 9 | 2 | 65 | 8 | NA | 0 | Pos |
| 10 | 2 | 22 | 8 | NA | 1+ | Pos |
| 11 | 2 | 24 | 8 | NA | 3+ | Neg |
| 12 | 3 | 17 | 7 | NA | 3+ | Pos |
| 13 | 2 | 34 | 8 | NA | 1+ | Neg |
| 14 | 3 | 23 | 8 | NA | 3+ | Neg |
| 15 | 1 | NA | 0 | 0 | NA | NA |
| 16 | 2 | 16 | 8 | NA | 2+FISH-ve | Neg |
| 17 | 2 | 14 | 8 | NA | 2+FISH+ve | Neg |
| 18 | 3 | 21 | 7 | NA | 1+ | Neg |
| 19 | 3 | 20 | 4 | 0 | 1+ | Neg |
| 20 | 2 | 26 | 8 | NA | 3+ | Pos |
| 21 | 2 | 53 | 8 | NA | 1+ | Pos |
| 22 | 3 | 20 | 3 | 0 | 3+ | Neg |
| 23 | NA | NA | 7 | NA | NA | Neg |
| 24 | 3 | 19 | 8 | NA | 2+FISH-ve | Pos |
| 25 | 2 | 49 | 0 | NA | 2+FISH-ve | Pos |
| 26 | 3 | 31 | 0 | 0 | 2+FISH-ve | Pos |
| 27 | 2 | 42 | 7 | NA | 0 | Neg |
| 28 | 3 | 47 | 0 | 0 | 1+ | Pos |
| 29 | 3 | 36 | 4 | NA | 0 | Neg |
| 30 | 3 | 25 | 2 | 0 | 2+FISH-ve | Pos |
| 31 | 3 | 22 | 8 | NA | 2+FISH-ve | Pos |
| 32 | 2 | 20 | 8 | NA | 2+FISH-ve | Neg |
| 33 | 3 | 18 | 2 | NA | 0 | Pos |
| 34 | 2 | 12 | 8 | NA | 2+FISH-ve | Neg |
| 35 | 3 | 37 | 4 | 0 | 0 | Neg |
| 36 | 2 | 21 | 8 | NA | 0 | Pos |
| 37 | 1 | 11 | 8 | NA | 0 | Neg |

**Supplementary Table S3 - Significantly differentially expressed genes between diagnostic and core biopsies**

| NIT 50 | Mean log2 Fold Change (EB/CB) |
|---|---|
| HBA2 | -1.23 |
| HBB | -1.17 |
| GOLGA6A | 0.08 |
| TMEM255B | 0.09 |
| LCA5L | 0.11 |
| C20orf141 | 0.11 |
| ZNF565 | 0.11 |
| LRCH1 | 0.14 |
| APOLD1 | 0.14 |
| ABL2 | 0.14 |
| FAM86FP | 0.15 |
| EDNRB | 0.15 |
| FLYWCH1 | 0.15 |
| ABCA6 | 0.16 |
| ITSN1 | 0.16 |
| WDFY2 | 0.16 |
| SPDYE3 | 0.18 |
| GOLGA8K | 0.19 |
| PTGS2 | 0.20 |
| KLF6 | 0.22 |
| RASA3 | 0.22 |
| SLC2A3P2 | 0.22 |
| ATF3 | 0.22 |
| GPR183 | 0.22 |
| KRTAP19-6 | 0.23 |
| NR4A3 | 0.24 |
| NPIPA3 | 0.24 |
| SIK1 | 0.25 |
| NR4A2 | 0.26 |
| ZSWIM4 | 0.30 |
| NRP1 | 0.31 |
| LAMB1 | 0.36 |
| SRGN | 0.36 |
| C8orf4 | 0.39 |
| SGK1 | 0.45 |
| EGR3 | 0.52 |
| GEM | 0.53 |
| SLC2A3 | 0.59 |
| RASD1 | 0.61 |
| MEG3 | 0.64 |
| JUN | 0.75 |

| | |
|---|---|
| RGS1 | 0.76 |
| NR4A1 | 0.77 |
| ZFP36 | 0.83 |
| CYR61 | 0.97 |
| RGS2 | 1.02 |
| FOS | 1.13 |
| EGR1 | 1.48 |
| DUSP1 | 1.56 |
| FOSB | 1.60 |

**Supplementary Table S4 - Cross-table comparing IHC subtypes and PAM50 subtype assignments in the diagnostic core biopsy samples**

| | **Basal** | **Her2** | **LumA** | **LumB** | **Normal** |
|---|---|---|---|---|---|
| **ER-/Her2-** | 0.21 | 0.07 | 0.43 | 0.00 | 0.29 |
| **ER+/Her2-** | 0.05 | 0.11 | 0.66 | 0.09 | 0.09 |
| **ER+/Her2+** | 0.25 | 0.13 | 0.56 | 0.06 | 0.00 |

## References


1. Desmedt, C. *et al.* Biological processes associated with breast cancer clinical outcome depend on the molecular subtypes. *Clin. Cancer Res.* **14,** 5158–65 (2008).
